# Development and validation of a nomogram to predict cancer-specific survival of mucinous epithelial ovarian cancer after cytoreductive surgery

**DOI:** 10.1186/s13048-023-01213-2

**Published:** 2023-06-27

**Authors:** Guanchen Ma, Shaoqing Zeng, Yingjun Zhao, Jianhua Chi, Li Wang, Qingshui Li, Jing Wang, Shuzhong Yao, Qi Zhou, Youguo Chen, Xiaofei Jiao, Xingyu Liu, Yang Yu, Yabing Huo, Ming Li, Zikun Peng, Ding Ma, Ting Hu, Qinglei Gao

**Affiliations:** 1 Department of Gynecological Oncology, Tongji Hospital, Tongji Medical College, Huazhong University of Science and Technology, 1095 Jiefang Ave, Wuhan, 430000 China; 2 National Clinical Research Center for Obstetrics and Gynecology, Cancer Biology Research Center (Key Laboratory of the Ministry of Education), Tongji Hospital, Tongji Medical College, Huazhong University of Science and Technology, Wuhan, China; 3 Department of Gynecology, Cancer Hospital of Zhengzhou University (Henan Tumor Hospital), Zhengzhou, China; 4 Department of Gynecologic Oncology, Shandong Cancer Hospital & Institute, Shandong, China; 5 Department of Gynecological Oncology, Affiliated Tumor Hospital of Xiangya School of Medicine, Central South University, Changsha, China; 6 Department of Obstetrics and Gynecology, The First Affiliated Hospital, Sun Yat-sen University, No. 58, the 2nd Zhongshan Road, Yuexiu District, Guangzhou, 510080 China; 7 Department of Gynecologic Oncology, Chongqing Cancer Hospital, Chongqing, 400030 China; 8 Department of Obstetrics and Gynecology, The First Affiliated Hospital of Soochow University, Suzhou, 215123 China

**Keywords:** Mucinous ovarian cancer, Nomogram, Cancer-specific survival, SEER database

## Abstract

**Background:**

Mucinous epithelial ovarian cancer (mEOC) is a relatively uncommon subtype of ovarian cancer with special prognostic features, but there is insufficient research in this area. This study aimed to develop a nomogram for the cancer-specific survival (CSS) of mEOC based on Surveillance, Epidemiology, and End Results (SEER) database and externally validate it in National Union of Real World Gynecological Oncology Research and Patient Management (NUWA) platform from China.

**Methods:**

Patients screened from SEER database were allocated into training and internal validation cohort in a ratio of 7: 3, with those from NUWA platform as an external validation cohort. Significant factors selected by Cox proportional hazard regression were applied to establish a nomogram for 3-year and 5-year CSS. The performance of nomogram was assessed by concordance index, calibration curves and Kaplan-Meier (K-M) curves.

**Results:**

The training cohort (n = 572) and internal validation cohort (n = 246) were filtered out from SEER database. The external validation cohort contained 186 patients. Baseline age, tumor stage, histopathological grade, lymph node metastasis and residual disease after primary surgery were significant risk factors (*p* < 0.05) and were included to develop the nomogram. The C-index of nomogram in training, internal validation and external validation cohort were 0.869 (95% confidence interval [CI], 0.838-0.900), 0.839 (95% CI, 0.787–0.891) and 0.800 (95% CI, 0.738–0.862), respectively. The calibration curves of 3-year and 5-year CSS in each cohort showed favorable agreement between prediction and observation. K-M curves of different risk groups displayed great discrimination.

**Conclusion:**

The discrimination and goodness of fit of the nomogram indicated its satisfactory predictive value for the CSS of mEOC in SEER database and external validation in China, which implies its potential application in different populations.

**Supplementary Information:**

The online version contains supplementary material available at 10.1186/s13048-023-01213-2.

## Introduction

Ovarian cancer is the eighth most common cancer in women worldwide, which causes more than 312,000 new cases and 206,000 deaths per year globally [1]. In all cases, epithelial ovarian cancer (EOC) occupies an enormous preponderance of morbidity and mortality. Among all subtypes of EOCs, mucinous epithelial ovarian cancer (mEOC) is relatively rare, with many misdiagnosed cases of extra-ovarian metastasis such as tumors of gastrointestinal origin [2]. As histopathological techniques develop, the incidence rate of primary mEOC has fallen from about 10% to 3–5% of all EOCs [3]. Apart from the low prevalence, mEOC displays different histological and biological features from other subtypes. When treated with debulking surgery followed by conventional platinum-based regimen, mEOC patients manifest inferior response rate and poorer prognosis than patients of other subtypes [4]. Besides, the prognosis of mEOC is very heterogeneous among different clinicopathological patients [5]. Previous studies revealed that several factors could influence the survival of mEOC, including age at diagnosis, stage of disease, histological grade, residual disease status, lymphovascular invasion, and so forth [6–9]. Among the above factors, advanced tumor stage takes up the most influential position. Most primary mEOC patients were diagnosed at an early stage and had good prognosis after debulking surgery. However, mEOC with International Federation of Gynecology and Obstetrics (FIGO) stage III/IV had a rather poor prognosis due to a worse response to subsequent treatment than other subtypes. The median overall survival of patients diagnosed at FIGO stage III/IV was only about 14.6 months according to the retrospective study [10]. Nowadays, prognosis predictive analysis specifically for mEOC is still insufficient, hence it’s meaningful to develop a practical and convenient model to offer the foresight of mEOC survival for both doctors and patients.

The Surveillance, Epidemiology, and End Results (SEER) database is a population-based cancer reporting system in specific geographic regions of the United States [11]. The SEER research data submitted from 17 registries in November 2019 covers approximately 26.5% of the US population [12]. Correspondingly, the National Union of Real World Gynecological Oncology Research and Patient Management (NUWA) Platform, established in 2019, is the first multi-center gynecological oncology research and disease management big data platform in China. As of the first quarter of 2022, NUWA platform has completed the data collection and structuring of medical records from 7 medical centers across the country, which gathers the widest range of information on gynecological tumors in China. The above data sources are both well representative, from which stable and reliable prediction results could be obtained. In this study, we aimed to construct and validate a nomogram for the cancer-specific survival (CSS) of mEOC after cytoreductive surgery based on SEER database and externally validate it in NUWA platform from China.

## Materials and methods

### Patients selection

All the information of patients who met the selection criteria was extracted by using the SEER*Stat Software Version 8.4.0 (https://seer.cancer.gov/seerstat/) and randomly allocated into the training cohort and internal validation cohort, in a ratio of 7: 3. The inclusion criteria were as follows: (1) histopathologically confirmed mEOC diagnosed between 2010 and 2019; (2) had received cytoreductive surgery during first-line treatment and known residual disease, (3) alive or dead due to cancer, (4) complete survival data. Patients with borderline tumors, history of other malignant tumors or missing values of any variable would be excluded. Likewise, the information of an external validation cohort was acquired from NUWA platform correspondingly. Each participating clinical center in NUWA platform had obtained approval of the institutional ethical review board. The study was approved by the Ethics Committee of Tongji Hospital of Huazhong University of Science and Technology and informed consent was waived (2020-S337).

### Covariates and outcome

We included several clinical and pathological factors into analysis, which were accessible in both SEER database and NUWA platform. Owing to different versions of tumor stage records in SEER database, we incorporated FIGO stage and SEER Summary Stage together in the analysis. The “Localized”, “Regional” and “Distant” of SEER Summary Stage corresponded to FIGO stage IA-IB, IC-IIB and III-IV, respectively [13]. Candidate variables were baseline age (years old) at diagnosis (≤ 50, > 50), marital status (married, other conditions), histopathological differentiation (well, moderately, poorly), tumor stage (FIGO IA-IB/ localized, IC-IIB/ regional, III-IV/ distant), serum CA125 (negative/ unknown, positive), lymph node metastasis (no/ no resection, yes) and residual disease after cytoreductive surgery (no residual disease [R0], residual disease < 1 cm [R1], residual disease ≥ 1 cm [R2]) [14]. The definition of positive CA125 was that the value of serum CA125 exceeded the upper limit of the reference value [15]. The main outcomes were 3-year and 5-year CSS of mEOC patients. CSS referred to the time from diagnosis to death caused by cancer or last follow-up.

### Statistical analysis

The chi-squared test was employed to compare the proportion of each categorical variable in the three cohorts. To evaluate the effect of different variables on CSS of mEOC patients, univariate and multivariate Cox proportional hazard regression analyses were performed in the training cohort. Variables that were statistically significant (*p* < 0.05) through univariate analysis would be subsequently included in multivariate analysis. Hazard ratio (HR) and 95% confidence interval (CI) were calculated to evaluate each variate. A nomogram for 3-year and 5-year CSS was further applied to visualize the effect of statistically significant variables (*p* < 0.05).

To assess the performance of nomogram, further detailed analyses were conducted. The concordance index (C-index) and receiver operating curves (ROC) of 3-year and 5-year CSS were applied to measure the nomogram’s predictive accuracy [16]. Calibration curves were used to evaluate the consistency between predicted and actual survival. Internal and external validation was conducted by bootstrap resampling in cohorts from SEER database and NUWA platform, respectively [17]. According to the total points of every patient in the training cohort, optimal cut-off points would be identified by X-tile software [18], by which patients in the three cohorts were divided into low, moderate and high-risk groups. Kaplan-Meier (K-M) curves were used to depict CSS between different risk groups of three cohorts.

Statistical analysis was conducted by SPSS version 26.0 (IBM Corp.) and R version 4.2.0 (www.R-project.org). *P*-value < 0.05 was considered statistically significant.

## Results

### Demographic and clinicopathological characteristics of patients

**Fig. 1:**
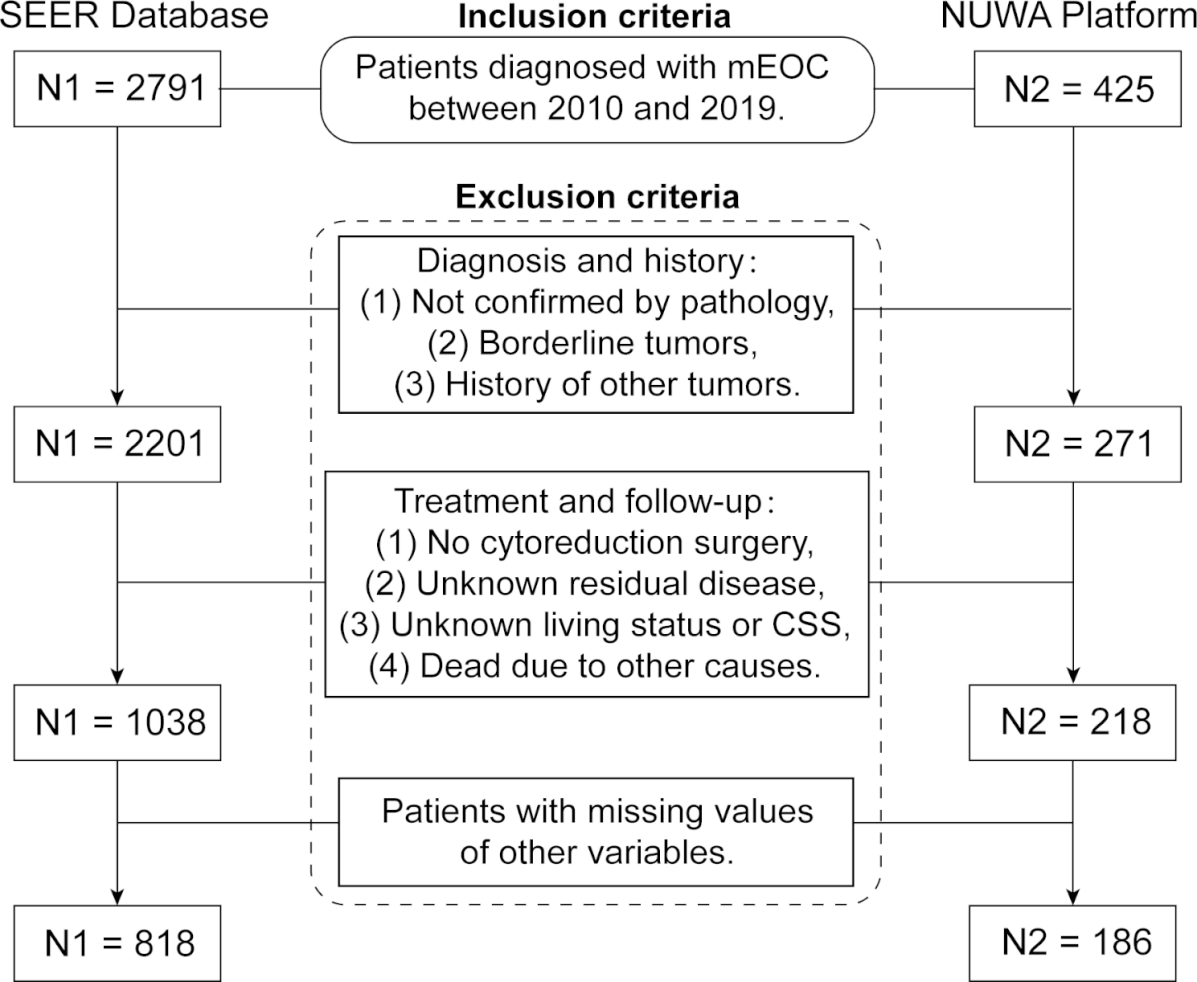
Flow chart of patient screening in the SEER database and NUWA platform

According to the inclusion criteria, a total of 818 patients were identified from SEER database, divided into the training cohort (n = 572) and internal validation cohort (n = 246) at random. Meanwhile, an external validation cohort consisting of 186 patients was enrolled from NUWA platform (Fig. 1). Missing values existed during data screening because information of this study was collected retrospectively, which were 220 patients (21.1%) in SEER Database and 32 (14.7%) patients in NUWA platform, respectively. We compared the characteristics and CSS of included patients with those of deleted patients in the two databases and found there was no difference in all the variables (Supplementary Table 1). Therefore deleting missing data wouldn’t affect the following analysis.

Patients who were diagnosed at older than 50 and other marital statuses accounted for a larger proportion in training (55.4%, 52.8%) and internal (53.3%, 52.8%) validation cohort compared with external validation cohort (41.9%, 13.4%; all *p* < 0.05). Additionally, in contrast to the external validation cohort, there were more patients with FIGO stage IA-IB/ localized stage (59.1%, 60.8%; *p* < 0.001), negative/ unknown serum CA125 (49.3%, 49.2%; *p* = 0.011) and no residual disease (90.4%, 91.5%, *p* < 0.001) in the two cohorts of SEER Database. Distribution of histopathological differentiation and lymph node metastasis had no significant difference among the three cohorts (*p* > 0.05). Detailed information is shown in Table 1.

**Table 1:** Clinicopathological characteristics of mucinous epithelial ovarian cancer patients in training and validation cohorts

Variable	Training cohort [n(%)]	Internal validation cohort [n(%)]	External validation cohort [n(%)]	*P*-value
**Age (yrs)**				0.006
≤ 50	255 (44.6%)	115 (46.7%)	108 (58.1%)	
> 50	317 (55.4%)	131 (53.3%)	78 (41.9%)	
**Marital status**				< 0.001
Married	270 (47.2%)	116 (47.2%)	161 (86.6%)	
Other conditions	302 (52.8%)	130 (52.8%)	25 (13.4%)	
**Differentiation**				0.217
Well	258 (45.1%)	107 (43.5%)	77 (41.4%)	
Moderately	234 (40.9%)	104 (42.3%)	70 (37.6%)	
Poorly	80 (14.0%)	35 (14.2%)	39 (21.0%)	
**Tumor stage**				< 0.001
IA-IB/ Localized	338 (59.1%)	148 (60.8%)	31 (16.7%)	
IC-IIB/ Regional	155 (27.1%)	64 (26.0%)	87 (46.8%)	
III-IV/ Distant	79 (13.8%)	34 (13.8%)	68 (36.6%)	
**CA125**				0.011
Negative/Unknown	282 (49.3%)	121 (49.2%)	69 (37.1%)	
Positive	290 (50.7%)	125 (50.8%)	117 (62.9%)	
**LNM**				0.105
No/No resection	556 (97.2%)	234 (95.1%)	175 (94.1%)	
Positive	16 (2.8%)	12 (4.9%)	11 (5.9%)	
**Residual Disease**				< 0.001
R0	517 (90.4%)	225 (91.5%)	129 (69.4%)	
R1	29 (5.1%)	11 (4.5%)	22 (11.8%)	
R2	26 (4.5%)	10 (4.1%)	35 (18.8%)	
**Total**	572 (100%)	246 (100%)	186 (100%)	

*LNM* lymph node metastasis, *R0* no residual disease, *R1* residual disease < 1 cm, *R2* residual disease ≥ 1 cm

### Variable selection and nomogram construction

**Fig. 2:**
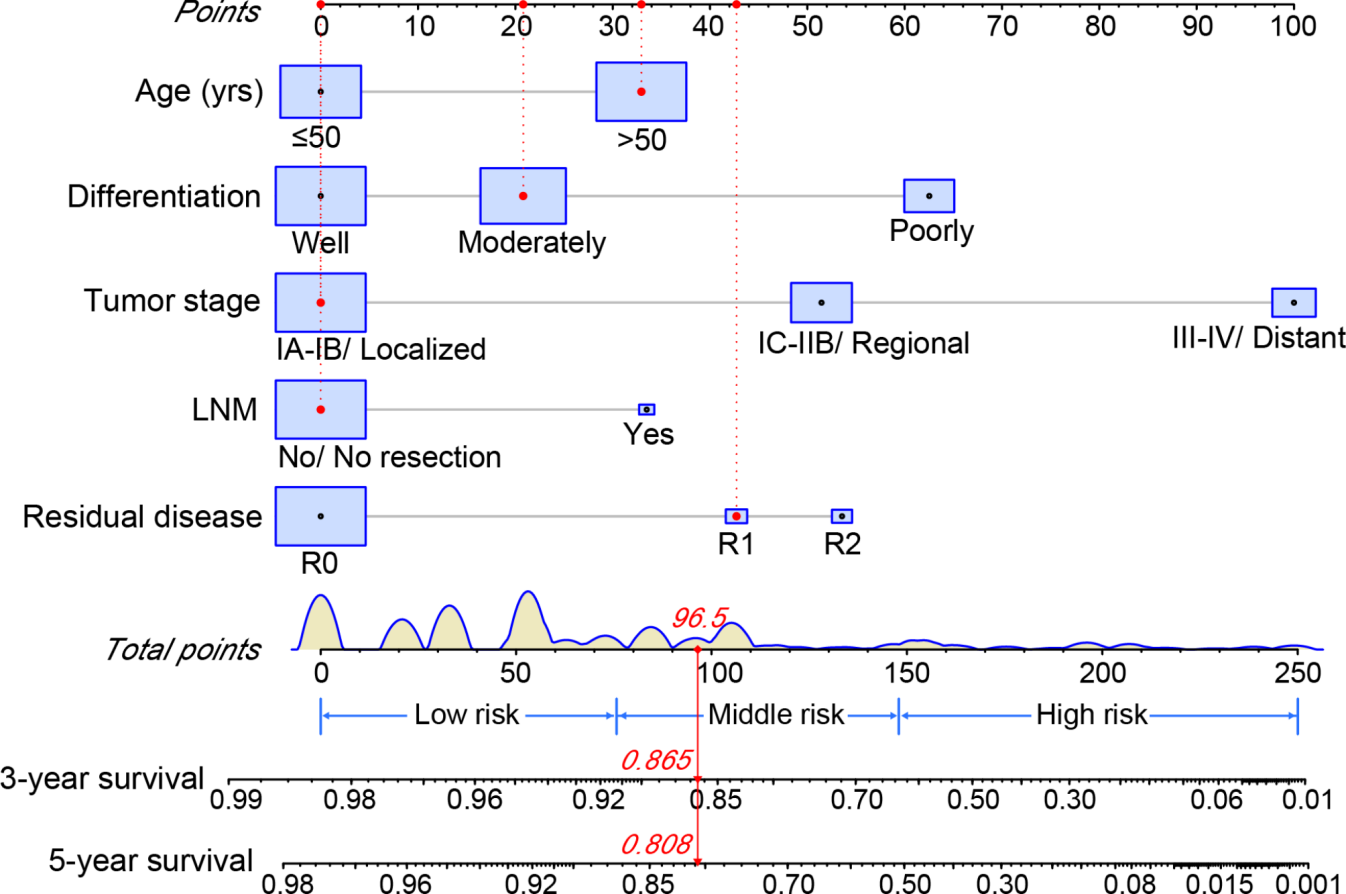
Nomogram for predicting 3-year and 5-year cancer-specific survival of mucinous epithelial ovarian cancer in SEER database. *FIGO* the International Federation of Gynecology and Obstetrics, *LNM* lymph node metastasis

The results of univariate and multivariate Cox regression analyses of predictors influencing CSS are summarized in Table 2. In the univariate Cox regression analysis, age at diagnosis, histopathological differentiation, serum CA125, tumor stage, lymph node metastasis and residual disease were significantly associated with the CSS of mEOC (*p* < 0.05). The independent prognostic factors were further demonstrated by multivariate Cox regression analysis, which were age at diagnosis, histopathological differentiation, tumor stage, lymph node metastasis and residual disease (*p* < 0.05). Based on the above prognostic factors, a nomogram was constructed to assess 3-year and 5-year CSS of mEOC patients (Fig. 2). Every patient was evaluated by each variable with a score ranging from 0 to 100. All the variable scores were added up to obtain total points, which could predict the corresponding CSS on the axis at the bottom of the nomogram.

**Table 2:** Univariate and multivariate Cox regression analysis of cancer-specific survival in the training cohort

Variable	Univariate Cox Regression			Multivariate Cox Regression		
	HR	95%CI	*P*-value	HR	95%CI	*P*-value
Age (yrs)						
≤ 50	Reference			Reference		
> 50	2.111	1.363–3.269	0.001	2.096	1.330–3.303	0.001
**Marital status**						
Married	Reference			Reference		
Other conditions	1.267	0.849–1.890	0.246	/	/	/
**Differentiation**			< 0.001			< 0.001
Well	Reference			Reference		
Moderately	2.349	1.370–4.028	0.002	1.565	0.902–2.715	0.111
Poorly	8.765	5.118–15.010	< 0.001	3.941	2.188–7.101	< 0.001
**CA125**						
Negative/Unknown	Reference			Reference		
Positive	2.252	1.473–3.445	< 0.001	1.227	0.788–1.911	0.364
**Tumor stage**			< 0.001			< 0.001
IA-IB/ Localized	Reference			Reference		< 0.001
IC-IIB/ Regional	3.204	1.781–5.762	< 0.001	3.075	1.701–5.560	< 0.001
III-IV/ Distant	23.103	13.599–39.250	< 0.001	8.720	4.574–16.626	< 0.001
**LNM**						
No/No resection	Reference			Reference		
Positive	6.560	3.576–12.033	< 0.001	2.009	1.028–3.923	0.041
**Residual Disease**						
R0	Reference			Reference		< 0.001
R1	7.263	4.180-12.619	< 0.001	2.536	1.360–4.728	0.003
R2	10.386	6.123–17.619	< 0.001	3.206	1.716–5.989	< 0.001

*LNM* lymph node metastasis, *R0* no residual disease, *R1* residual disease < 1 cm, *R2* residual disease ≥ 1 cm

### Evaluation of the nomogram’s performance

**Fig. 3:**
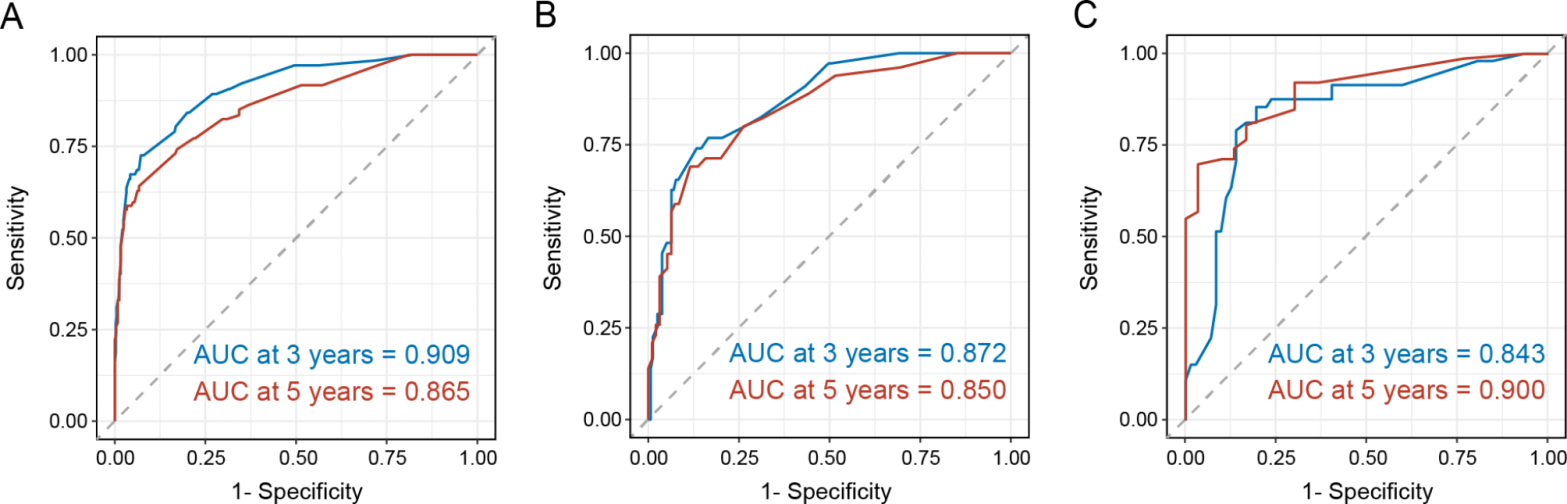
Receiver operating curves for 3-year and 5-year cancer-specific survival in the **(A)** training cohort, **(B)** internal validation cohort and **(C)** external validation cohort. *AUC* area under the curve

**Fig. 4:**
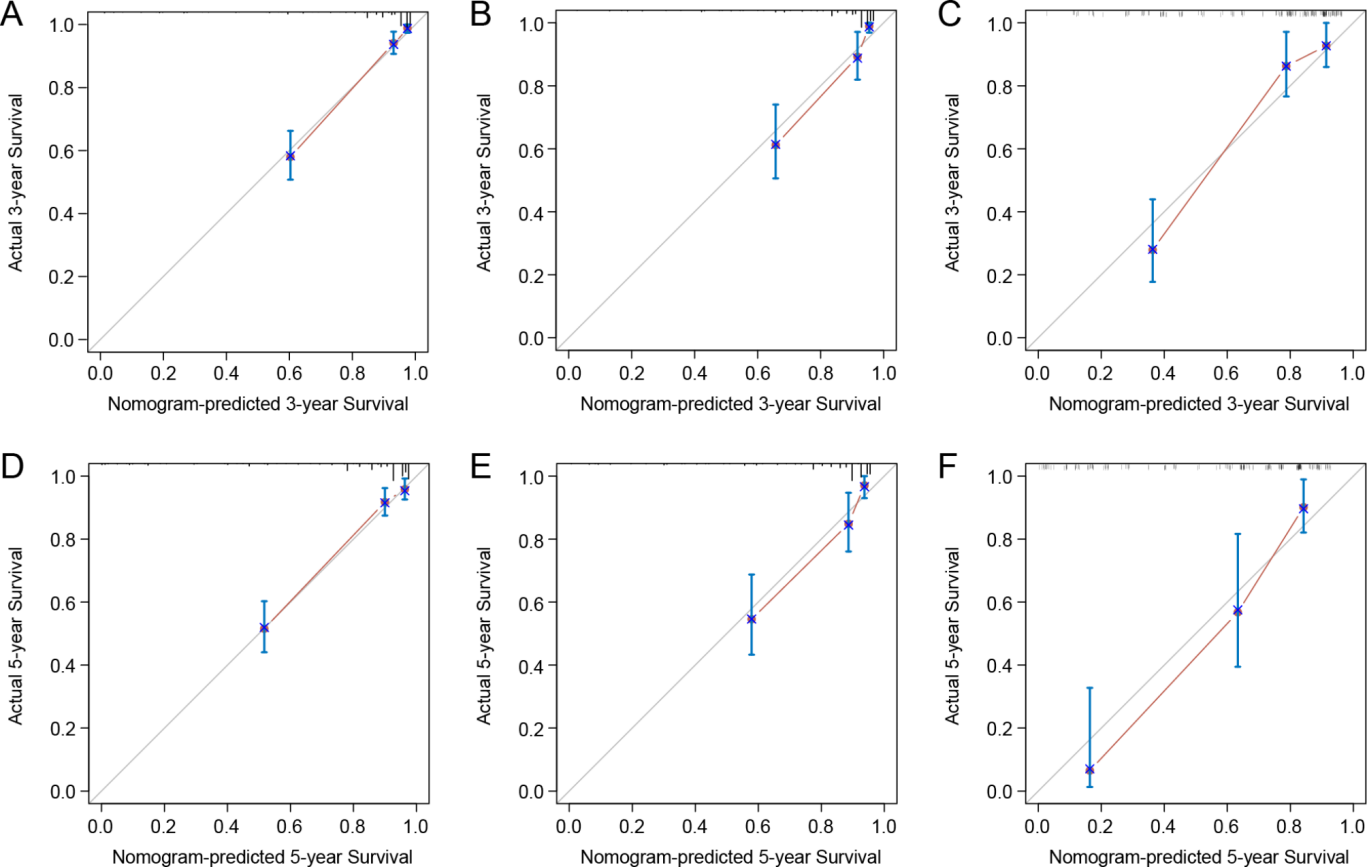
Calibration curves for 3-year, 5-year cancer-specific survival in the **(A, D)** training cohort, **(B, E)** internal validation cohort, and **(C, F)** external validation cohort

The C-index of nomogram in training cohort, internal validation cohort and external validation cohort were 0.869 (95% CI, 0.838-0.900), 0.839 (95% CI, 0.787–0.891) and 0.800 (95% CI, 0.738–0.862), respectively. The ROC of 3-year and 5-year CSS in Fig. 3 depicted superb discrimination of the nomogram, with the area under the curve (AUC) of 3-year CSS of patients in the three cohorts reaching 0.909, 0.872 and 0.843. Correspondingly, the AUC of 5-year CSS of patients in the three cohorts were 0.865, 0.850, and 0.900, respectively. The calibration curves indicated good consistency between the nomogram-predicted CSS and the actual CSS in the training and internal validation cohort, which was relatively inferior in the external validation cohort (Fig. 4).

**Fig. 5:**
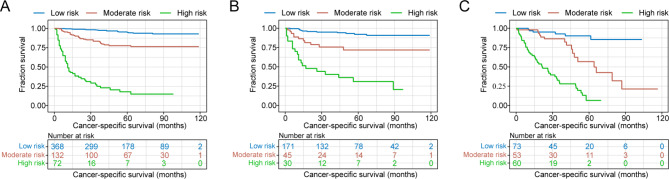
Kaplan-Meier curves for low, moderate, high risk groups in **(A)** training cohort, **(B)** internal validation cohort and **(C)** external validation cohort

By using the X-tile software, optimal cut-off points for total points of each patient in the training cohort were identified, which were 75.7 and 147.9 (Supplementary Fig. 1). Therefore, patients in each cohort was stratified into low risk (total points ≤ 75.7), moderate risk (75.7 < total points ≤ 147.9) and high risk (total points > 147.9) group. The K-M curves for CSS showed favorable discrimination between each risk group in the three cohorts respectively (Fig. 5). The median CSS was not reached at the cut-off time of data collection in low-risk groups of the three cohorts and moderate-risk group of the training and internal validation cohort. The median CSS of the moderate-risk group in the external validation cohort was 64.2 months. For high-risk groups, the median CSS was 11.0, 17.0 and 21.3 months in training, internal validation and external validation cohort. The 5-year CSS rates of low, moderate and high-risk group were 94.8%, 77.7%, and 17.8% in the training cohort, which were 92.1%, 71.7% and 30.9% in the internal validation cohort. For low, moderate and high-risk group in external validation cohort, 5-year CSS rates were 90.1%, 56.8%, 6.5%, respectively.

## Discussion

As an uncommon subtype of ovarian cancer, mEOC is always confusingly mixed with other histotypes in prognosis analysis. There were scarcely any studies or subgroup analysis exclusive for mEOC, which may obscure its independent importance. M. Gore et al. [19] conducted a multi-center phase III clinical trial (mEOC/GOG 0241) to study the effect of different regimens on the prognosis of advanced-stage mEOC, but the high rate of misdiagnosis and slow accrual led to the failure of the research. Recently, a few retrospective survival prediction studies specialized in mEOC came into view, which may compensated for the deficiency of prospective studies. Yang et al. [20] constructed a prognostic model for overall survival (OS) and CSS of mucinous adenocarcinoma based on SEER database, which included baseline age, race, TNM stage, grade, CA125 and chemotherapy in the nomogram. The C-index of nomogram for OS was 0.845 and that for CSS was 0.862. Richardson et al. [8] focused on patients with FIGO stage I mEOC in the National Cancer Database of the United States. They utilized factors such as baseline age, grade, sub-stage, lymphovascular invasion and presence of malignant ascites to develop a model to predict 5-year and 10-year survival.

Compared to previous studies, we included mEOC patients diagnosed between 2010 and 2019 in SEER database and NUWA platform, which were relatively newly diagnosed and had long enough follow-up period and relatively higher quality of data in this study. Since primary mEOC was rather difficult to be distinguished from mucinous carcinomas metastatic to the ovary previously [2, 21]. With the development of ancillary diagnostic techniques, the proportion of misdiagnosed cases may be lower in the recent decade than before. What’s more, the data of variables such as residual disease were more accessible after 2010 in the above two databases. In the phase of model building, we analyzed available factors in SEER database that might influence CSS. Consistent with previous studies, baseline factors such as older age at diagnosis, higher stage of disease, poorer histopathological differentiation and lymph node metastasis had detrimental impacts on CSS [6, 9, 22, 23]. We integrated the FIGO stage and SEER summary stage into analysis instead of the TNM stage included in previous studies [20, 24], which took into account both specificity in the staging of ovarian cancer and applicability in SEER database. Notably, we included residual disease into the survival prediction model of mEOC for the first time and demonstrated that the less residual disease after cytoreductive surgery was, the less risk of death would be, whereas other models hadn’t focused on it [8, 20, 24]. It is widely acknowledged that optimal debulking plays a prominent role in the prognosis of all histotypes including mEOC because it reduces the probability of recurrence and lays a good foundation for subsequent treatment [25, 26]. Both prospective and retrospective studies of the association between cytoreduction surgery and EOCs came to similar conclusion [27–29]. With regard to the evaluation of our nomogram, the C-index, calibration curves and K-M curves showed satisfactory discrimination and accuracy in three cohorts. Based on the cut-off points of patients in the training cohort, patients in each cohort could be divided into high, medium and low-risk groups, thereby their CSS could be clearly stratified to provide auxiliary information for the following treatment. Patients with high risk of cancer-specific death should receive more intensive attention during follow-up. Remarkably, there were certain differences among high-risk groups of the three cohorts, which may be owing to certain differences in data distribution of variables between SEER database and NUWA platform, as well as inherent differences between American and Chinese patients. Although there were more patients in external validation cohort diagnosed at advanced stage, they were all treated in large tertiary hospitals with high-quality medical services, hence the mCSS of high-risk group in external validation cohort was longer than those of training and internal validation cohorts. On the other hand, since advanced tumor stage was the most influential variable in the nomogram, after 5 years of follow-up, most patients in the high-risk group of external validation cohort were dead because of high lethality of advanced mEOC, which resulted in lower 5-year CSS compared with training and internal validation cohorts. Certainly, we considered that the relatively small sample size of the external validation cohort was also one of the reasons, thus we would expand the size of dataset to improve sample representativeness in the future. To our knowledge, this is the first study to concentrate on postoperative mEOC patients exclusively. Secondly, since there hasn’t been any predictive model for mEOC evaluated in Chinese patients so far [8, 20, 24], our study could make up for the scarcity of relevant research and broaden its applicability in different ethnic groups. In general, this nomogram may help doctors and patients to predict the survival of mEOC immediately after cytoreductive surgery, so that they can make appropriate decisions for more personalized clinical management.

Nevertheless, this study had several limitations. Firstly, the accuracy and discrimination of the nomogram were relatively inferior in the external validation cohort. It could be acceptable because there is an incongruous distribution of data between SEER Database and NUWA platform [16]. Finding an external validation cohort that was consistent with the composition ratio of each variable in the training set was quite difficult. The inherent difference among various racial groups and different medical conditions between countries may also contribute to differences in tumor development and prognosis [30]. Secondly, owing to retrospective data collection of our study, missing values seemed inevitable during data acquisition. Whereas, we didn’t found significant difference among all the characteristics and CSS within the two databases. Therefore we considered deleting patients with missing values and complete case analysis wouldn’t bring much selection bias to statistical result. In addition, more information on diagnosis and treatment procedures should be taken into consideration when selecting predictors for nomogram, such as more other blood tumor markers [31], germline or somatic genetic testing [32, 33], detailed chemotherapy [23, 34], and so on. Regrettably, data of variables mentioned above is still not collected or available in SEER Database so far. The above infrequently recorded factors are counting on public databases to collect more diverse information in the future. Finally, from a broader perspective, we look forward to validating the nomogram in other databases or prospective cohorts to improve its stability and practicability.

## Conclusions

In summary, this study analyzed several risk factors and found that older baseline age, advanced tumor stage, poorer histopathological differentiation, lymph node metastasis and larger residual disease were independent predictors for CSS of mEOC patients. The nomogram constructed from the above factors based on SEER database was firstly validated in NUWA platform from China, which exhibited good performance and practicability in patients of different races. Whereas, it also needed further improvements.

## Electronic supplementary material

Below is the link to the electronic supplementary material.

**Supplementary Fig. 1** Identification of optimal cut-off points for total score of patients in the training cohort.

**Supplementary Table 1** Comparison of characteristics of included patients and patients with missing values in SEER Database and NUWA Platform

## Data Availability

All data generated or analyzed during this study are included in this published article [and its supplementary information files].

## Abbreviations

AUC: Area under the curve
CI: Confidence interval
CSS: Cancer-specific survival
EOC: Epithelial ovarian cancer
FIGO: International Federation of Gynecology and Obstetrics
HR: Hazard ratio
K-M: Kaplan-Meier
mEOC: Mucinous epithelial ovarian cancer
NUWA: National Union of Real World Gynecological Oncology Research and Patient Management
OS: Overall survival
ROC: Receiver operating curve
SEER: Surveillance, Epidemiology, and End Results
