# Supplementary figures and images for: Development and validation of a nomogram to predict cancer-specific survival of mucinous epithelial ovarian cancer after cytoreductive surgery
Source: J Ovarian Res. 2023 Jun 27;16:120. doi: 10.1186/s13048-023-01213-2 (PMC10294480; doi:10.1186/s13048-023-01213-2)

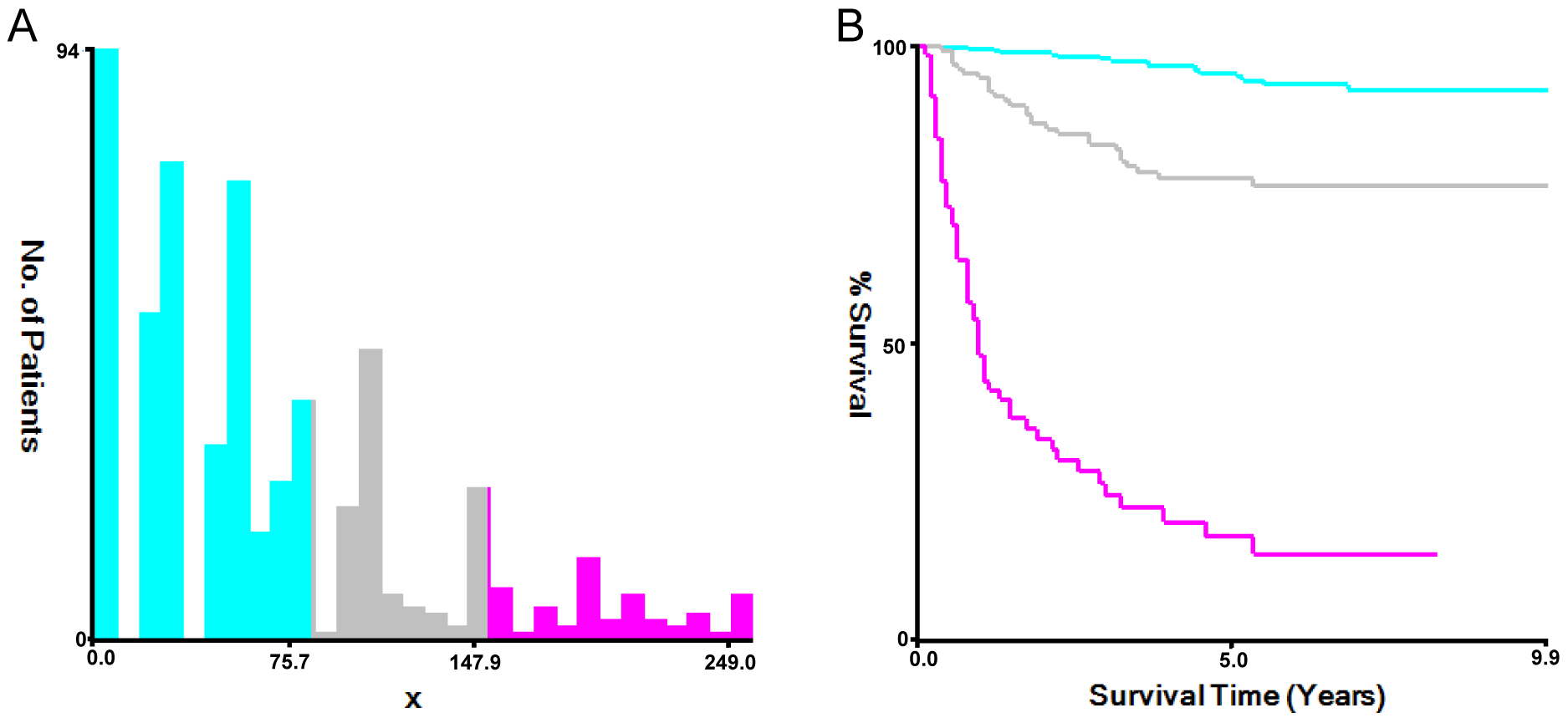

Supplement: Supplementary file 1 — Supplementary Fig. 1 Identification of optimal cut-off points for total score of patients in the training cohort. [file 13048_2023_1213_MOESM1_ESM.tif]
