# Supplementary material for: Development and validation of a nomogram to predict cancer-specific survival of mucinous epithelial ovarian cancer after cytoreductive surgery
Source: J Ovarian Res. 2023 Jun 27;16:120. doi: 10.1186/s13048-023-01213-2 (PMC10294480; doi:10.1186/s13048-023-01213-2)
Supplement: Supplementary file 2 — Supplementary Table 1 Comparison of characteristics of included patients and patients with missing values in SEER Database and NUWA Platform [file 13048_2023_1213_MOESM2_ESM.docx]

**Supplementary Table 1** Comparison of characteristics of included patients and patients with missing values in SEER Database and NUWA Platform.

| **Variable** | **SEER Database** | | | **NUWA Platform** | | |
| --- | --- | --- | --- | --- | --- | --- |
|  | **Inclusion**  **[n(%)]** | **Missing**  **[n(%)]** | ***P*-value** | **Inclusion**  **[n(%)]** | **Missing**  **[n(%)]** | ***P*-value** |
| **Age (yrs)** |  |  | 0.560 |  |  | 1.000 |
| ≤50 | 370 (45.2%) | 105 (47.7%) |  | 108 (58.1%) | 19 (59.4%) |  |
| >50 | 448 (54.8%) | 115 (52.3%) |  | 78 (41.9%) | 13 (40.6%) |  |
| **Marital status** |  |  | 0.433 |  |  | 0.109 |
| Married | 386 (47.2%) | 111 (50.5%) |  | 161 (86.6%) | 24 (75.0%) |  |
| Other conditions | 432 (52.8%) | 109 (49.5%) |  | 25 (13.4%) | 8 (25.0%) |  |
| **Differentiation** |  |  | 0.871 |  |  | 1.000 |
| Well | 365 (44.6%) | 3 (50.0%) |  | 77 (41.4%) | 2 (50.0%) |  |
| Moderately | 338 (41.3%) | 3 (50.0%) |  | 70 (37.6%) | 1 (25.0%) |  |
| Poorly | 115 (14.1%) | 0 (0.0%) |  | 39 (21.0%) | 1 (25.0%) |  |
| **Tumor stage** |  |  | 0.131 |  |  | 0.260 |
| IA-IB/ Localized | 486 (59.4%) | 123 (58.3%) |  | 31 (16.7%) | 8 (29.6%) |  |
| IC-IIB/ Regional | 219 (26.8%) | 48 (22.7%) |  | 87 (46.8%) | 10 (37.0%) |  |
| III-IV/ Distant | 113 (13.8%) | 40 (19.0%) |  | 68 (36.6%) | 9 (33.3%) |  |
| **CA125** |  |  | 0.491 |  |  | 0.768 |
| Negative/Unknown | 403 (49.3%) | 102 (46.4%) |  | 69 (37.1%) | 11 (34.4%) |  |
| Positive | 415 (50.7%) | 118 (53.6%) |  | 117 (62.9%) | 21 (65.6%) |  |
| **LNM** |  |  | 0.518 |  |  | 0.371 |
| No/No resection | 790 (96.6%) | 215 (97.7%) |  | 175 (94.1%) | 31 (100%) |  |
| Positive | 28 (3.42%) | 5 (2.27%) |  | 11 (5.91%) | 0 (0.00%) |  |
| **Residual Disease** |  |  | 0.532 |  |  | 0.697 |
| R0 | 742 (90.7%) | 194 (88.2%) |  | 129 (69.4%) | 20 (62.5%) |  |
| R1 | 40 (4.89%) | 14 (6.4%) |  | 22 (11.8%) | 5 (15.6%) |  |
| R2 | 36 (4.40%) | 12 (5.45%) |  | 35 (18.8%) | 7 (21.9%) |  |
| **mCSS [95% CI]** | Not reached | Not reached | 0.866 | 64.2  [43.7, 84.70] | 62.0  [38.3, 85.70] | 0.651 |

*LNM* lymph node metastasis, *R0* no residual disease, *R1* residual disease < 1cm, *R2* residual disease ≥ 1cm, *mCSS* median cancer-specific survival.
